# Incidence rate of Rift Valley fever exposure in humans and livestock from a longitudinal study in Northern Kenya

**DOI:** 10.1038/s41598-025-24693-2

**Published:** 2025-11-20

**Authors:** Mathew Muturi, Athman Mwatondo, Ard M. Nijhof, Richard Nyamota, Daniel Nthiwa, Kristina Roesel, Lilian Wambua, James Akoko, S. M. Thumbi, Bernard Bett

**Affiliations:** 1 Freie Universität Berlin, Institute of Parasitology and Tropical Veterinary Medicine, Robert-von-Ostertag-Str. 7-13, 14163 Berlin, Germany; 2 International Livestock Research Institute, Nairobi, Kenya; 3 Kenya Zoonotic Disease Unit, State Directorate of Veterinary Services, Kenya National Public Health Institute, Nairobi, Kenya; 4 Centre for Epidemiological Modelling and Analysis, University of Nairobi, Nairobi, Kenya; 5 Department of Medical Microbiology and Immunology, University of Nairobi, Nairobi, Kenya; 6 Veterinary Centre for Resistance Research, Freie Universität Berlin, Berlin, Germany; 7 Institute for Parasitology and Tropical Veterinary Medicine, Freie Universität Berlin, Berlin, Germany; 8 Paul G Allen School for Global Health, Washington State University, Pullman, WA USA; 9 Department of Biological Sciences, University of Embu, Embu, Kenya; 10 Department of Animal Breeding and Husbandry in the Tropics and Subtropics, University of Hohenheim, Stuttgart, Germany; 11 Institute for Immunology and Infection Research, University of Edinburgh, Edinburgh, Scotland, UK

**Keywords:** Rift Valley fever, Incidence, Humans, Livestock, Kenya, Virus-host interactions, Risk factors, Viral infection

## Abstract

Unravelling the mechanisms of Rift Valley fever virus (RVFV) maintenance in endemic areas during interepidemic periods is critical for enhancing early detection and response. Unfortunately, data on key epidemiological parameters, such as incidence rates, which are crucial for risk assessments and designing targeted interventions, are almost nonexistent. We conducted a longitudinal study of 1,938 pastoral livestock and 814 livestock keepers in an endemic region of northern Kenya from March 2022 to May 2023 to estimate the incidence rate of RVFV exposure and determine risk factors for infection. We assessed exposure to RVFV in humans and livestock using an anti-RVF immunoglobulin enzyme-linked immunosorbent assay. RVFV incidence was calculated in livestock and humans as the number of new seroconversions over the total animal and person time at risk, respectively. An interval-censored regression model was employed to compute the baseline hazard and identify risk factors. We observed 113 new livestock infections over 805 animal-years at risk, translating to an annual livestock incidence rate of 0.14 per animal-year (95% CI: 0.12–0.17). Multivariable analysis found species, acaricide use, and period of sampling were significant factors that influence RVFV incidence in livestock. In humans, 15 RVFV seroconversions were observed over 629 person-years at risk, yielding an incidence rate of 24 per 1000 person-years (95% CI: 13–39). Age and sex were not significant predictors of RVFV human exposure. Seroconversion in livestock and humans suggests that low-level transmission between vertebrate hosts and vectors could be the primary mechanism for RVF viral persistence in endemic areas. Our findings highlight the need for routine serosurveillance and continuous public health education on RVF infection and prevention during interepidemic periods.

## Introduction

Rift Valley fever, a zoonotic mosquito-borne viral disease caused by a *Phlebovirus* in the family *Phenuviridae*, is most prevalent in East Africa’s Great Rift region, where the virus (RVFV) was first identified in 1931^1,]^^2^. The disease has also been detected and is believed to be enzootic in various regions of Africa and the Arabian Peninsula^3,4^. Outbreaks have historically been confined to these areas, but the spread and persistence of the virus in new territories, including the Global North, poses a viable threat due to the presence of competent vectors in these regions^5^.

RVF is primarily transmitted to animals through mosquito vectors, with at least 30 species across eight genera believed to have RVFV transmission potential^2,6^. Aedine mosquitoes are thought to play a crucial role in initiating transmission during the early stages of the epidemic phase, while other mosquito genera, like *Culex*, are believed to play a vital role in propagating infection when the population of *Aedes* mosquitoes recedes in the latter stages of outbreaks^6,7^. A range of other arthropods, like phlebotomine sandflies, *Culicoides* biting midges, and ticks, have been identified as possible mechanical vectors of RVFV under experimental conditions, but their role in nature remains unascertained^8,9^.

In livestock, RVF majorly presents with non-specific symptoms such as inappetence, debilitation, and diarrhoea; however, the classical syndromes indicative of an outbreak are mass abortions affecting all stages of pregnancy and high perinatal mortality rates^10^. Humans primarily get infected through contact with infected animals or their tissues and fluids, although mosquito-borne transmission is also plausible but rare^11^. Human cases majorly present with constitutional symptoms, but one to two percent of cases advance to a more severe form that clinically varies depending on the system affected. Ocular, hepatic, neurologic, and hemorrhagic disease and death are common manifestations of severe RVF in humans^12^.

The epidemiology of RVF is complex and multifaceted. A confluence of ecological abiotic and biotic factors, intrinsic host factors such as immunity, and human practices such as migration and irrigation are all known to influence RVFV emergence^13^. However, the mechanisms of how these factors interact are poorly understood. While it is generally agreed that outbreaks are linked to weather events that support vector amplification and increased transmission, there is debate on how and where RVFV is maintained during the decades-long interepidemic periods (IEP) that precede outbreaks^14,15^. A pioneering theory suggests that the virus could be maintained through vertical transmission in *Aedes* eggs, which remain buried and viable for years until the right ecological conditions trigger the hatching of RVFV-infected progeny^16,17^. Although this is the principal theory cited in literature, limited data supports the same^15,18^. An alternative theory is that the virus is maintained primarily through a low-level mosquito-host infection cycle that causes sub-clinical disease in susceptible ruminant livestock and wildlife^11,19^. Evidence of acute livestock, human, and wildlife infections in endemic and non-endemic regions of Africa during IEPs strongly supports this theory^20,21^. However, it remains unclear whether either of these mechanisms is sufficient to maintain RVFV endemicity independently^22^. Unravelling the epidemiological complexity around RVFV maintenance requires answering questions on livestock and human incidence rates and the risk factors for infection during IEPs. Unfortunately, most of the available data are limited to cross-sectional serological studies and routine passive surveillance data, which lack the causal insights needed to elucidate maintenance and transmission dynamics of RVFV in endemic environments. As a result, the endemicity of RVF is not in question, but data on population-level incidence, which is key to detecting cryptic transmission rates during IEPs, are almost nonexistent^23,24^. Incidence rates, defined as the proportion of new cases or events occurring over a specified period in a population at risk, are an important measure of morbidity. Incidence rates provide a direct estimate of the risk of disease spread among susceptible individuals, which is critical in understanding disease transmission dynamics, identifying populations at highest risk, assessing temporal trends in transmission, and evaluating interventions^25,26^.

Nearly a century since RVF was reported in Kenya, this paper presents the first estimates of human and livestock RVF incidence rates, providing evidence of interepidemic transmission in humans and livestock. The findings underscore the need for sustained RVF surveillance and interventions, even during interepidemic periods.

## Materials and methods

### Ethics statement

#### Human study

The study was authorized by the Kenya National Commission for Science, Technology & Innovation (NACOSTI/P/21/14359), and ethical approval for the human study was granted by the International Livestock Research Institute (ILRI) Institutional Research Ethics Committee (ILRI-IREC2020-07). All adult participants provided written informed consent for human sampling. Children between 13 years and 17 years provided written assent after parental/guardian permission was obtained, while for those below 13 years, only written parental/guardian permission was required.

#### Animal study

Livestock sampling was approved by the ILRI Institutional Animal Care and Use Committee (ILRI-IACUC 2021-18/1). The study was also approved by the Kenya National Director of Veterinary Services. Household heads provided written consent before livestock sampling. The animal study adheres to the Animal Research Reporting of In Vivo Experiments (ARRIVE) guidelines.

**Accordance statement** - All human studies were carried out in accordance with the relevant guidelines. All animal procedures were also conducted in accordance with the protocols outlined in the IACUC approval. The animals used in the study remained with their owners throughout the study period and continued under the care of their owners after the study.

## Study site

**Fig. 1:**
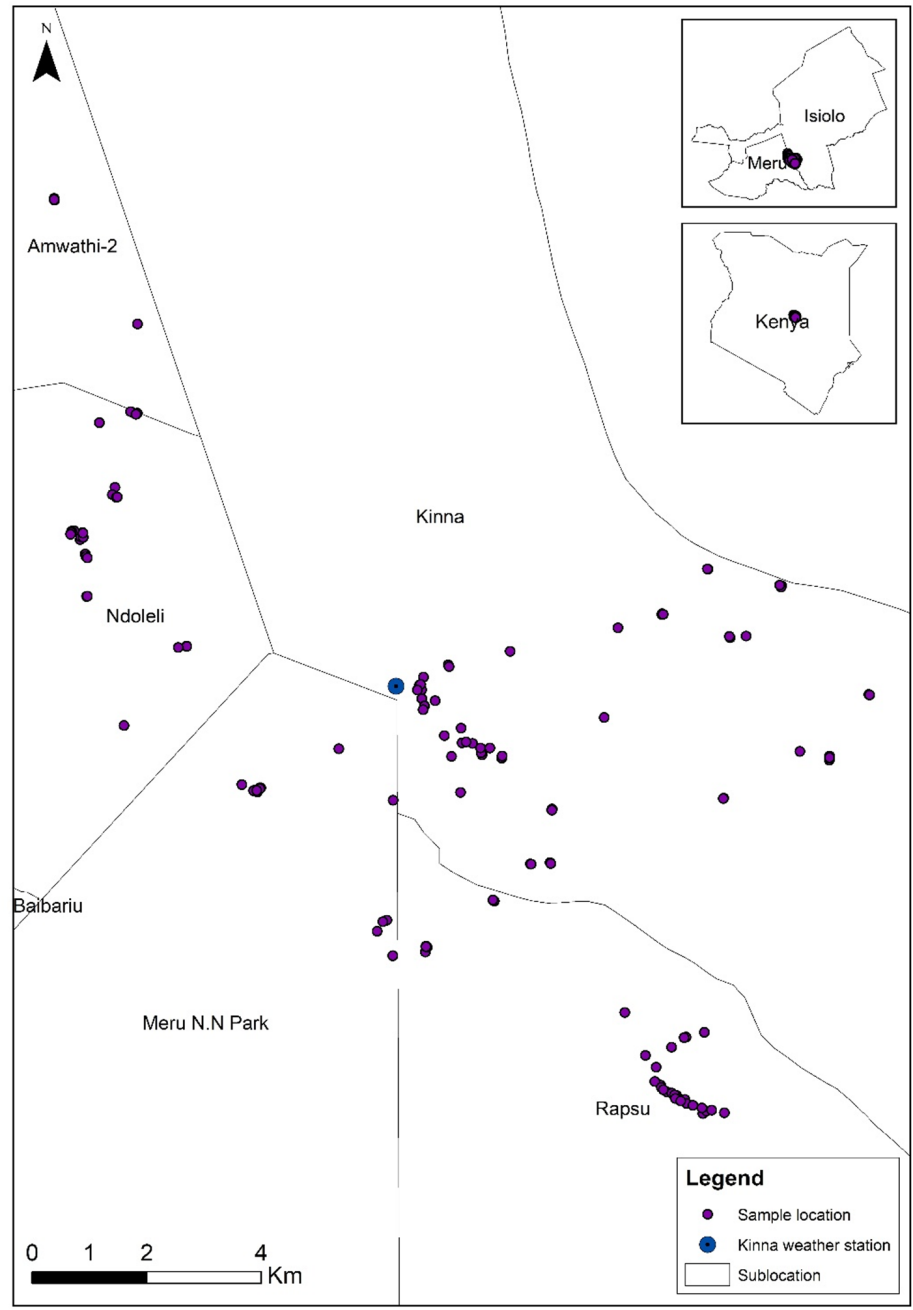
Map of the study area: Kinna village and distribution of randomly sampled households. The map was generated using QGIS version 3.40. The shapefile used to create the map was obtained from https://gadm.org/download_country.html.

The study was conducted in Kinna village (approximately 20 km^2^), Garbatulla Sub-County, Isiolo County, between March 2022 and May 2023 (Fig. 1). The study site is considered a high-risk region for RVF due to past outbreaks and high seroprevalence in humans and livestock. Our previous baseline study in the same area reported a RVF prevalence of 21.7% (95% CI: 19.9–23.7%) in humans and 28.4% (95% CI: 24.9–31.9%) in livestock^27^. The study site is a semi-arid, low-lying area where nomadic pastoralism, characterised by the movement of large herds of cattle, sheep, and goats in search of water and pasture, and communal livestock grazing serves as the economic mainstay. The mean annual temperature is 26 °C, and the area receives an average rainfall of 580 mm annually^28^.

## Sample size determination and study design

The study was a one-year cohort study to estimate the incidence rate of livestock (cattle, sheep, and goats) and humans. We calculated the sample size using the formula$\:n=Z^2/d^2$ to estimate the incidence rate with 10% precision and 95% confidence^29^. The naive sample sizes were multiplied by a variance inflation factor (design effect) to account for variance in precision and to maintain statistical power due to expected substantial clustering of observations at the herd and household levels. In livestock, the design effect, *D*, was calculated using the formula$\:\:D=1+\left(m-1\right)p$, where *m* represents the number of animals recruited from a herd (the average cluster size) and $\:p$ is the intracluster correlation coefficient. A cluster size of 15 was used while the $\:p$ factor was assumed to be 0.2, which is the highest value for most infectious livestock diseases^30,31^. The sample size was then adjusted by 50% to account for projected losses to follow-up, giving a minimum sample size of 1,612 animals. For the human sample size, a design effect of 1.8 was used, assuming five household members will be sampled, and a $\:p$ factor of 0.2, which is a conservative estimate to ensure the sample size is not underestimated. This gave a minimum sample size of 637 humans.

### Selection and recruitment of the livestock and human cohort

As this was a household-based longitudinal study, we enrolled animals that were owned and managed by consenting livestock farmers in the study area. No animals were purchased, relocated, or removed from their usual management systems. We used two-tier inclusion criteria to identify and recruit study animals (cattle, sheep, and goats) from participating herds. We first developed a sampling frame of livestock-owning households in each village, using information from village elders on the number of species and livestock owned. Households were then randomly selected from this sampling frame. Once a household was identified, livestock owners identified healthy animals older than three months, with no history of RVF vaccination or RVF-like illness in the six months preceding the study. RVF-like illness was defined as the presence of at least one of the following syndromes: abortion, unexplained bleeding, weight loss, lethargy, or other signs of generalised illness of unknown aetiology. Second, livestock owners were asked to identify animals they were likely to keep for the study duration to minimise losses to follow-up. At least 15 animals (five per species) were randomly selected from those that met the inclusion criteria in each herd. The selected animals were clinically examined to confirm their health status and then tagged with a pre-printed, colour-coded ear tag. A blood sample was collected and transported to the International Livestock Research Institute (ILRI) for screening for anti-RVFV antibodies.

Human participants were eligible if they had lived in a household within the study area for at least six months before the study. A household was defined as individuals sharing a common cooking area. All household members, except children under two years, were recruited into the study upon consenting. Each recruited household member was assigned a unique identifier, after which a demographic and risk factor questionnaire was administered and a blood sample collected. All human and livestock samples were processed and transported for storage at −20 °C, followed by subsequent testing at ILRI. Our previous study provides a detailed description of the sampling, transport, and storage procedures^27^. All data was collected and managed electronically through the Open Data Kit (ODK) database systems version 1.30.1.

## Follow-up of recruited humans and livestock and identification of incidence cases

Humans and livestock were recruited in two phases to achieve the desired sample size: from March 18 to April 4, 2022, and from July 7 to 15, 2022. The recruited human and livestock study subjects were visited for repeat sampling every three to four months for one year. A questionnaire was administered during each visit to collect household and subject-level demographic and putative risk factor information. The first repeat sampling was conducted between 24 June 2022 and 6 July 2022, while the second was conducted between 16 November 2022 and 26 November 2022. The final sampling was done between 3 March 2023 and 5 May 2023; the extended sampling period was due to the inaccessibility of some study animals due to flooding.

## Laboratory procedures

All livestock and human samples were tested at ILRI for anti-RVFV Immunoglobulin G (IgG) antibodies using a multi-species competitive enzyme-linked immunosorbent assay (c-ELISA) from IDvet (Innovative Diagnostics, Grabels, France). The multispecies kit was selected due to high validity values; specificity of 0.986 (0.971–0.998 95% CI) and sensitivity of 0.854 (0.655–0.991 95% CI)^32^. The IDvet kit has previously been used for RVF serology in humans^33,34^. In brief, 100 µL of diluted serum samples and kit positive and negative controls were loaded onto precoated microtiter plates and incubated at 37 °C for 1 h. The plates were then washed three times using FCM wash buffer (1X), followed by adding 100 µL 1X conjugate solution. The reaction was incubated at room temperature for 30 min and then washed three times. The substrate solution (100 µL) was added to each well, and the test plate was incubated at room temperature for 15 min. The reaction was stopped by adding 100 µL of the stop solution, and the optical densities (ODs) were read at 450 nm wavelength using a BioTek ELISA reader (Synergy HT, United States). The sample competition percent (S/N%) was determined by dividing the mean OD of each sample by the mean OD for the negative control and multiplying by 100%. A sample was considered positive if S/N% was ≤ 40%, negative if > 50%, and borderline if the value was between 40 and 50% as per the manufacturer’s recommended cut-off values. The samples with borderline results were retested. Validation of all the test results was ensured through duplicate testing of all samples and controls. An incident case was defined as seroconversion from c-ELISA seronegative at baseline to seropositive during the follow-up period.

### Data analysis

Data analysis was done using R^®^ version 4.4.1^35^. After data merging and cleaning, initial descriptive statistical analyses included estimation of humans and livestock RVF seroprevalence using the *gmodels* package^36^. Livestock RVFV prevalence was tabulated for each categorical variable. In livestock, the categorical variables considered included animal sex (male and female), species (camels, goats, and sheep), and age (less than one year and above one year). The above descriptive statistical analyses were further calculated for each livestock species. Human seroprevalence estimates were also stratified by demographic characteristics. The Chi-square (χ^2^) test was used to assess the relationships between categorical variables and the seropositivity of RVF IgG. Samples that remained borderline after retesting were removed from the analysis.

To estimate incidence, we used an incidence-density analytic design, suitable for open human and livestock cohorts^37^. Pastoralist populations often experience a high loss to follow-up due to migration. As a result, recruited humans and animals have varying follow-up periods. In this approach, the number of new cases is divided by the total animal/person time at-risk to obtain an incidence rate. We first excluded cases that tested positive at the time of recruitment, ensuring that only RVFV seronegative subjects were eligible for follow up. When seroconversion was observed between two sampling periods, the later sampling date was designated as the estimated time of exposure. The duration at risk (in days) was calculated for each subject and then combined to determine the overall incidence of RVF exposure. We used the Poisson exact test to estimate the crude incidence rate with 95% confidence intervals.

### Risk-factor analysis

Survival analysis was used to identify risk factors for exposure. The follow-up data were treated as interval-censored because screening for infection was done every three to four months, and the exact time a study subject was infected was unknown. As such, the Interval-censored Regression (IcenReg) model for interval-censored survival data analysis in R was used for univariable and multivariable analysis to identify risk factors. The survival object used in this algorithm was specified using the following three variables:

1. Two characters indicating the start and end of the interval (upper and lower).
2. Boolean variable to identify the infection status.
3. Character variable specifying the type of censoring, which in this case was “interval2”.

We analysed multiple potential predictors of exposure in humans and livestock, including age, sex, sampling period, herd size, an observed increase in mosquito population in the last three months and acaricide use.

Univariable models were fitted for each independent variable to identify candidates for multivariable analysis. Variables with a p-value < 0.05 in the univariable analysis were included in the multivariable model. Hazard rate ratio, an expression of the hazard or chance of events occurring in the infected group as a ratio of the hazard of the events occurring in the non-infected group, was used to express risk. A hazard ratio of 1 implies no difference in risk between the infected and non-infected groups. A hazard ratio greater than one implies that the risk is higher in the exposed group and vice versa.

## Results

A total of 1,943 livestock, 689 cattle (35.4%), 631 goats (32.5%), and 623 sheep (32.1%) from 140 herds were recruited. The majority (84.5%) were female. The majority of sampled animals were over 12 months old (44.4%), followed by weaners (6 to 12 months old) at 38.2%. The remaining 17.3% were young animals aged between three and six months.

We enrolled 814 individuals from 140 households; the median household size was five (range: 1–16), with most participants (59.1%) being male. The median age was 22 years (Range: 2–90). Most of the study participants were involved directly or indirectly in livestock-keeping activities, with only 2.7% earning their livelihoods exclusively from off-farm activities. A total of 530 participants (65.1%) were recruited during the March-April period, while 284 (34.9%) were recruited during the July 2022 period. For livestock, 1,521 (78.3%) were recruited in March and April 2022, while 422 (21.7%) were recruited in July 2022.

### Sero-positivity during the baseline phase

The baseline seropositivity of RVF in livestock was 28.9% (95% CI: 26.9%−31.1%). Exposure was highest in cattle, at 31.7% (95% CI 28.2% − 35.3%), followed by goats at 28.8% (95% CI 25.3% − 32.4%) and sheep at 25.9% (95% CI: 22.5% − 29.4%), but these differences in seropositivity across species were not statistically significant. There was, however, a significant difference in positivity between different age categories, with older animals in all three species having higher positivity (χ² = 64.94; *P* = 0.001). No significant difference was observed in positivity between male and female livestock across species (χ² = 1.801; *P* = 0.17).

The human baseline seroprevalence was 7.4% (95% CI 5.8–9.2%) in the baseline survey. Differences in prevalence between different age groups and sexes were not statistically significant.

### Incidence rates

#### Livestock

We observed 113 new infections over 805.29 animal-years, translating to an annual livestock incidence rate of 0.14 per animal-year (95% CI: 0.12–0.17). Fifty-eight seroconversions were detected in cattle observed over 286.3 animal-years, yielding an incidence rate of 0.2 (95% CI: 0.15–0.26). Goats had the second-highest incidence rate, with 36 seroconversions over 267.7 animal-years, equating to an incidence rate of 0.14 (95% CI: 0.1–0.19). We recorded 19 seroconversions in sheep over 259.3 animal-years, translating to an annual incidence rate of 0.07 (95% CI: 0.04–0.11).

### Human incidence

We detected 15 human seroconversions over 629 person-years at-risk, giving an incidence rate of 24 per 1000 person-years (95% CI: 13–39).

### Risk factor analysis

Multiple predictors of human exposure to RVFV were analysed, but none were significant on univariable analysis (Table 1).

**Table 1:** Univariable analysis of select human RVFV exposure predictors.

Variable	Category	Hazard ratio	Std. error	*P*-value
Sex	Female	0.489	0.584	0.217
	Male	1*	-	-
Age in years	Age	1.013	0.012	0.236
Handling aborted material		2.1584	1.0357	0.458

*Reference value.

Multivariable analysis found species, acaricide use, and period of sampling were significant factors that influence RVFV incidence in livestock. Animal age and sex did not affect the incidence rate of RVF (Table 2). Cattle had a significantly higher hazard rate of RVF than sheep, but it was not significantly different from that of goats. RVF hazard was highest in period 1 (March to July 2022) compared to periods 2 (July to November 2022) and 3 (November 2022 to May 2023). Animals from herds where acaricides were used had a 34% lower risk of RVF infection.

**Table 2:** Factors associated with the incidence rate of RVFV seroconversion in livestock in Northern Kenya between March 2022 and May 2023.

Variable	Category	Exp (Est)	Std. error	*P* - value
Sex	Female	1*	-	-
	Male	1.015	0.414	0.971
Species	Bovine	1*	-	-
	Caprine	0.667	0.2959	0.17
	Ovine	0.311	0.3135	0.001
Period of sampling	Period 1	1*	-	-
	Period 2	0.447	0.2299	0.001
	Period 3	0.113	0.4279	0.001
Animal age	Livestock months	1.001	0.003635	0.681
Vector control	No acaricide use	1*	-	-
Acaricide use	Acaricide use	0.667	0.1469	0.006

*Reference value.

## Discussion

Our data provides empirical evidence of interepidemic RVF seroconversion in humans and unvaccinated livestock and, to our knowledge, represents the first report of RVF human incidence rate from a field study. This study detected interepidemic RVFV seroconversion across all three livestock species in the absence of an outbreak, strongly supporting the proposition that cryptic transmission in ruminant livestock could serve as the primary mechanism for RVF persistence in endemic regions. The data also provides evidence of cryptic transmission in humans, underlying the need to include RVF as a differential diagnosis for human febrile illnesses in endemic areas even during perceived low-risk periods.

The high seropositivity rates in the baseline survey used to identify naïve animals for follow-up were not unexpected. Serological studies in similar pastoral settings have reported comparable exposure rates, reflecting the endemicity of RVFV in northern Kenya^27,38,39^. The region’s high ambient temperatures, seasonal anomalous precipitation, low elevation, vast bushlands, high livestock density, and frequent livestock movement provide a suitable ecological niche for mosquito vector proliferation, sustaining a stable cycle of infected vector and naïve livestock host interaction that drives the high exposure rates^18,40,41^. The significant association between livestock age and seropositivity in the baseline survey further reaffirms this, as it implies cumulative RVFV exposure over time^23,42^. The high seropositivity rates likely underestimate the true seroprevalence because the estimates were derived from a sample of healthy and subclinical animals. On the flip side, the human prevalence detected is discernibly lower than that reported by similar studies in the same region^27,28,43^. The variance could be explained by the variability of RVF exposure due to the heterogeneity of environmental factors that affect transmission over time and space, for example, weather and vegetation, and possibly the influence of human behaviour on infection^44^.

The study detected interepidemic RVFV seroconversion across all three livestock species in the absence of an observed epizootic. This was evidenced by the lack of reports of RVF-like syndromes, such as mass abortions, idiopathic haemorrhages, or high perinatal mortality rates, in our livestock cohort, which was under active surveillance. This finding conforms with other studies that have reported interepidemic RVF transmission in sub-Saharan Africa^45–47^. Scientific opinion on the role of sub-clinical RVFV transmission in long-term RVFV maintenance varies significantly^14,15,18^. Our data strongly support the proposition that cryptic transmission in ruminant livestock could serve as the primary mechanism for RVF persistence in endemic regions. Although the incidence rate is one of the key parameters describing the transmission dynamics of vector-borne diseases, RVF incidence rate studies are few and far between^48,49^. We could not find a study that reports incidence rates from a follow-up survey in East Africa; however, a similar follow-up study in South Africa reported an incidence rate of 0.59 per animal-year (95% CI: 0.46–0.75) and 0.41 (95% CI 0.25–0.64) in cattle and goats sampled between June 2016 and June 2018 ^50^. While we note the incidence rates quoted are higher than our results, differences in sampling design, locality, and study period limit the comparison of the incidence rates. However, an interesting finding in both studies was that cattle had a significantly higher incidence rate than other species^50^. Another study reporting incidence rate estimates from cross-sectional studies in livestock in Tanzania also reported higher incidence rates in cattle^33^. The heterogeneity of RVFV incidence among hosts could indicate varying vector host preference; studies in Kenya and South Africa have shown that mosquito vectors feed more on cattle than sheep and goats, or could be attributed to biological determinants of susceptibility, or the effect of higher mobility rates in cattle, which exacerbate the risk of infection^51–53^.

Interestingly, livestock age was not a significant predictor. This suggests that viral exposure in animals aged three months and above occurs at the same rate or that age does not influence RVFV mechanistic correlates of protection in animals above three months. The paucity of data on RVFV immune responses and the complexities of intrinsic and extrinsic factors influencing vector-host preference urge caution in making specific inferences and highlight the need for further research on this topic^54,55^.

The highest rate of infection was noted in the 1 st sampling done between 24/06/2022 and 07/07/2022. While we cannot explicitly deduce what could explain the amplification of viral transmission because there were no marked differences in weather patterns, increased animal movement due to drought could be a plausible theory. Animal movement has been identified as a primary driver of endemic infections in Sub-Saharan Africa and one of the anthropogenic factors that support endemic circulation^21,40^. We did not account for animal movement as one of our independent parameters, but this is highly recommended for future prospective studies. Livestock in herds where the owners reported routine use of acaricides had a lower incidence. While this is unsurprising, it provides empirical evidence that supports vector-control programs as a critical pillar in RVF prevention and control, which has also been proven by other studies^13,56^.

To the best of our knowledge, this is the first report of human RVF incidence rates from a follow-up study in an endemic area. The detection of seroconversion in the human population indicates active RVF transmission during a period previously considered low-risk. This underscores the need to consider RVF as a differential diagnosis for undifferentiated fevers in endemic areas, even outside of outbreaks. Given the potential severity of RVF infections, continued public health interventions are essential, particularly enhanced surveillance and public education on vector control and infection prevention. None of the predictors were statistically significant in the risk factor analysis, likely due to the small number of incident cases during the follow-up period, which limited statistical power.

Our findings show that the incidence of RVF is moderately high in the study region because of endemic viral circulation. This implies that the risk of explosive outbreaks is high when ecological conditions that support vector proliferation, such as sustained flooding, are at play. This highlights the critical importance of adopting risk-based surveillance strategies that integrate monitoring of environmental indicators (e.g., number of rainfall days, vegetation changes) and sentinel herd sero-monitoring to improve the sensitivity of traditional passive surveillance systems that are based on clinical syndrome reporting^57,58^. Our data also reaffirms the need for sustained vector control and livestock vaccination even in non-outbreak periods. Further research is required to understand vector dynamics in the region and the ecological factors influencing periodic and species-specific variations in incidence in livestock. This study provides a baseline for human incidence rates. More systematic cohort studies in humans in different ecologies are needed to provide data on the predictors of human incidence to better inform mitigation measures.

Nearly a century since RVF was identified in the country, this is the first linked study to report field-based estimations on the incidence rate of RVFV in humans and livestock in Kenya, highlighting the critical need for implementing RVF mitigation measures even during interepidemic periods. Our study had some limitations. It is important to note the diagnostic limitations of using ELISA instead of a confirmatory Plaque Reduction Neutralisation (PRNT) test to rule out false positives and cross-immunoreactivity with other viruses. We therefore highly recommend the use of PRNT in future studies. Loss of follow-up was also a constraint, but this was accounted for in the study design and analysis.

## Data Availability

The data that support the findings of this study are available from the corresponding author upon reasonable request.

## Declarations

### Data analysis and interpretation

Mathew Muturi, Daniel Nthiwa, Bernard Bett, SM Thumbi.

### Manuscript review and approval

Mathew Muturi, Athman Mwatondo, Ard M. Nijhof, James Akoko, Daniel Nthiwa, Lilian Wambua, Kristina Roesel, SM Thumbi, Bernard Bett.
